# 16S rRNA Sequencing Analysis of the Gut Microbiota in Broiler Chickens Prophylactically Administered with Antimicrobial Agents

**DOI:** 10.3390/antibiotics10020146

**Published:** 2021-02-02

**Authors:** Matteo Cuccato, Selene Rubiola, Diana Giannuzzi, Elena Grego, Paola Pregel, Sara Divari, Francesca Tiziana Cannizzo

**Affiliations:** 1 Department of Veterinary Science, University of Turin, Grugliasco, 10095 Turin, Italy; matteo.cuccato@unito.it (M.C.); selene.rubiola@unito.it (S.R.); elena.grego@unito.it (E.G.); paola.pregel@unito.it (P.P.); tiziana.cannizzo@unito.it (F.T.C.); 2 Department of Agronomy, Food, Natural Resources, Animals and Environment, University of Padua, 35020 Legnaro, Italy; diana.giannuzzi@unipd.it

**Keywords:** prophylaxis, coccidiostat, broiler, 16S rRNA sequencing, ileum, caecum

## Abstract

In poultry production, gut microbiota (GM) plays a pivotal role and influences different host functions related to the efficiency of production performances. Antimicrobial (AM) use is one of the main factors affecting GM composition and functions. Although several studies have focused their attention on the role of AMs as growth promoters in the modulation of GM in broilers, the consequences of higher AM concentrations administered during prophylactic treatments need to be better elucidated. For this purpose, 16S rRNA gene sequencing was performed to evaluate the impact of different prophylactic AM protocols on the composition and diversity of the broiler GM. Diversity analysis has shown that AM treatment significantly affects alpha diversity in ileum and beta diversity in both ileum and caecum. In ileal samples, the *Enterobacteriaceae* family has been shown to be particularly affected by AM treatments. AMs have been demonstrated to affect GM composition in broiler. These findings indicate that withdrawal periods were not enough for the restoral of the original GM. Further studies are needed for a better elucidation of the negative effects caused by an altered GM in broilers.

## 1. Introduction

The intestinal microbiota is a complex microbial community that has established a symbiotic relationship with its animal host [1]. It is well known that gut microbiota (GM) plays an essential role as a first-line defense in the intestinal mucosal barrier [2]. This microbial community has evolved together with the animal host and through the colonization resistance ensures the prevention of animal health from the arousal of intestinal infectious disease [2,3]. In poultry production, GM plays a pivotal role and its several functions have been related to the efficiency of production performances [4,5,6]. Furthermore, GM influences different host functions, e.g., digestion of nutrients, immunity, and metabolism [1,7]. It has been demonstrated that GM perturbations could negatively affect intestinal health and lead to severe health consequences for animals, but also to economic losses for the farmer [7,8,9]. Antimicrobial (AM) use is one of the main factors affecting GM composition and functions. In recent years, there has been an increasing concern about antimicrobials resistance (AMR) and the role of AM use in the spreading of AMR, and the selection of multi-drug-resistant bacteria [10,11]. On the other hand, AM use leads to long-lasting disturbances of the commensal GM and may negatively affect broiler physiology [12]. Moreover, the broiler GM varies among the different intestinal tracts and its composition reflects their different functions [7]. Several studies focused their attention on the role of AMs as growth promoters in the modulation of GM in broilers [13,14,15], but the consequences of higher AM concentration on GM, e.g., during prophylactic treatments, need to be better elucidated. Therefore, the main aim of this study was to evaluate the impact of different prophylactic AM protocols on the composition and diversity of the broiler GM by 16S rRNA gene sequencing technology. In addition, we evaluated the different impact of AM treatment on the ileum and caecum microbiota.

## 2. Results

### 2.1. 16S rRNA Diversity Analysis

A total of 84 samples were analyzed both for ileal (*n* = 42) and caecal (*n* = 42) microbiota by 16S rRNA sequencing. A total of 8,036,623 raw reads (2 × 300 bp) were obtained after sequencing, with an average value of 95,674 reads/sample. After joint and quality filtering, a total of 4,963,263 reads passed the filters applied through the DADA2 plugin in the QIIME2 software package (https://qiime2.org; version: 2019.10), with an average value of 95,361 reads/sample. Following primer trimming and raw read quality filtering, ileal and caecal samples were analyzed separately. In order to avoid biases due to different sequencing depths, ileal samples and caecal samples were rarefied at 31,100 reads and 5600 reads, respectively; one sample from ileum was excluded from further analyses due to a low read count. After assigning taxonomies, a total of 3421 unique operational taxonomic units (OTUs) at 99% nucleotide sequence identity were identified in caecum samples, while 1153 unique OTUs were identified in ileum samples.

Alpha diversity analysis has shown that AM treatments have different consequences on ileum and caecum microbiota. Considering caecal samples, Pielou’s evenness (H = 5.38; *p* = 0.5) and Faith’s phylogenetic diversity (H = 7.69; *p* = 0.26) indicated no significant differences between samples. Instead, ileum samples were characterized by alpha diversity metrics statistically different with Pielou’s evenness (H = 13.53; *p* = 0.03) and Faith’s phylogenetic diversity (H = 13.12; *p* = 0.04).

**Figure 1:**
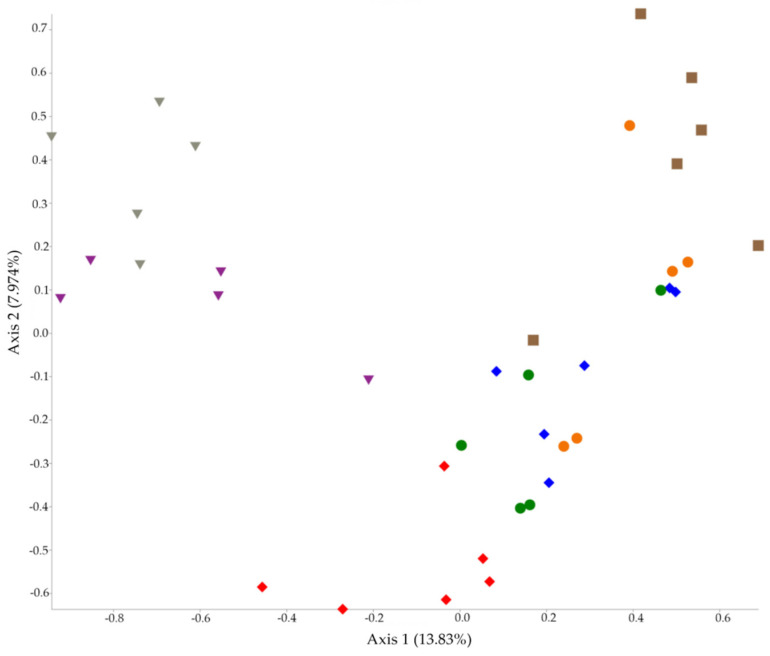
PCoA plot of beta diversity analysis of caecal samples. Amoxicillin group (AMX) (red), amoxicillin + diclazuril group (AMX + DCZ) (blue), thiamphenicol group (THP) (purple), thiamphenicol + diclazuril group (THP + DCZ) (grey), sulfadiazine + trimethoprim group (TRIM) (brown), diclazuril group (DCZ) (orange) and control group (K) (green).

**Figure 2:**
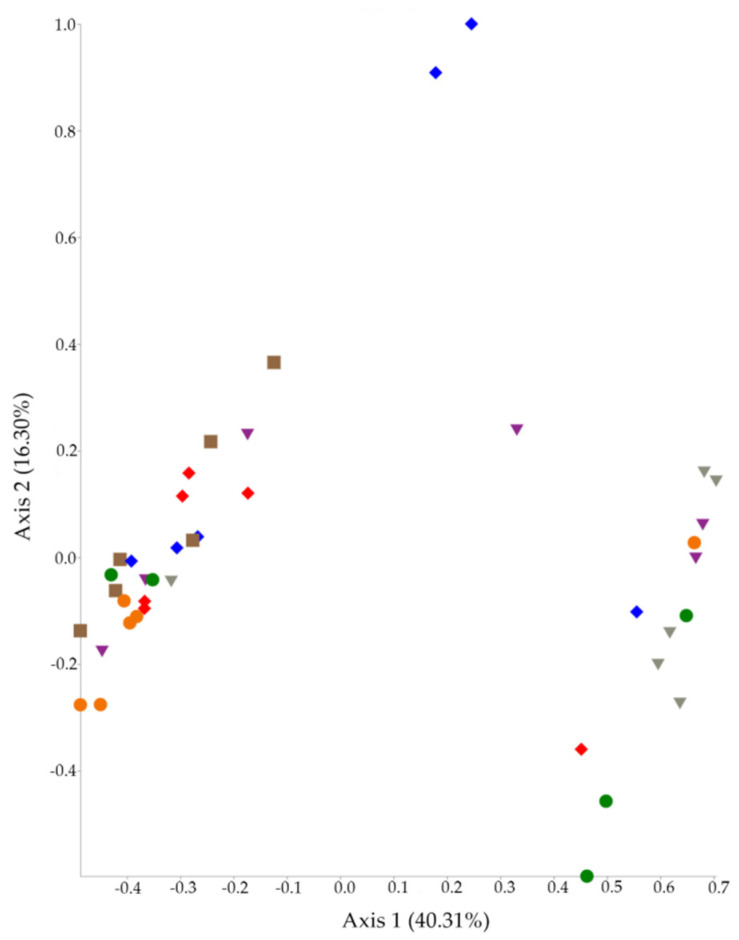
PCoA plot of beta diversity analysis of ileal samples. Amoxicillin group (AMX) (red), amoxicillin + diclazuril group (AMX + DCZ) (blue), thiamphenicol group (THP) (purple), thiamphenicol + diclazuril group (THP + DCZ) (grey), sulfadiazine + trimethoprim group (TRIM) (brown), diclazuril group (DCZ) (orange) and control group (K) (green).

In addition, the Permutational Multivariate Analysis of Variance (PERMANOVA) test has shown that beta diversity metrics were always statistically different between groups (*p* = 0.001 for caecum and *p* = 0.002 for ileum). Principal Coordinate Analysis (PCoA) plots were generated both for caecum and ileum using beta diversity metrics and are presented in Figure 1 (caecum) and Figure 2 (ileum).

### 2.2. 16S rRNA Taxonomy Analysis

**Figure 3:**
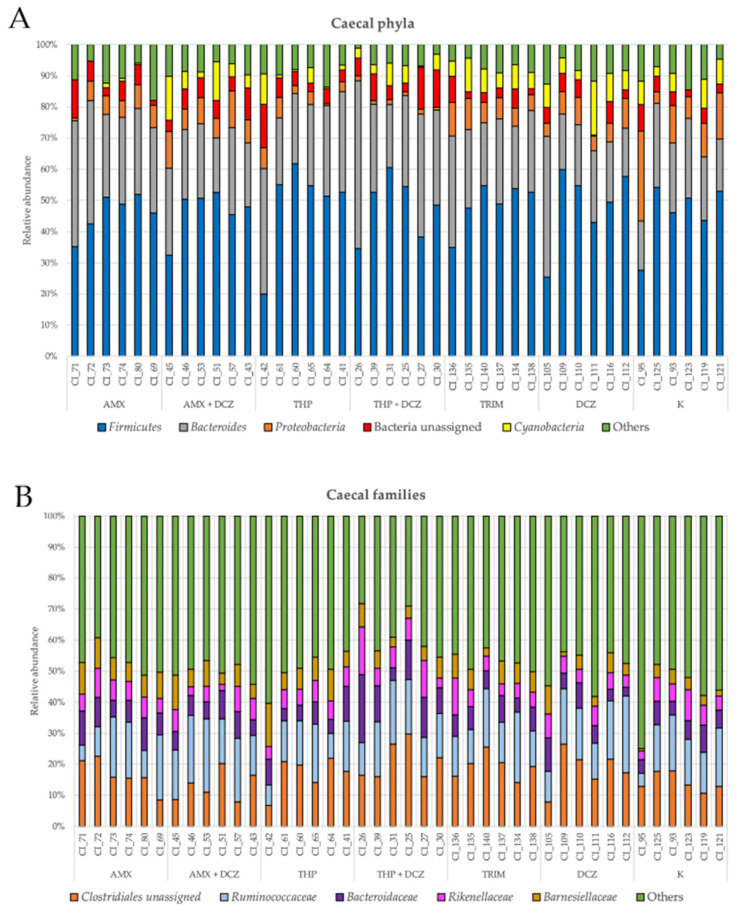
Relative abundance at the phylum (**A**) and family level (**B**) in caecum. Specific legends are reported for each bar plot.

**Figure 4:**
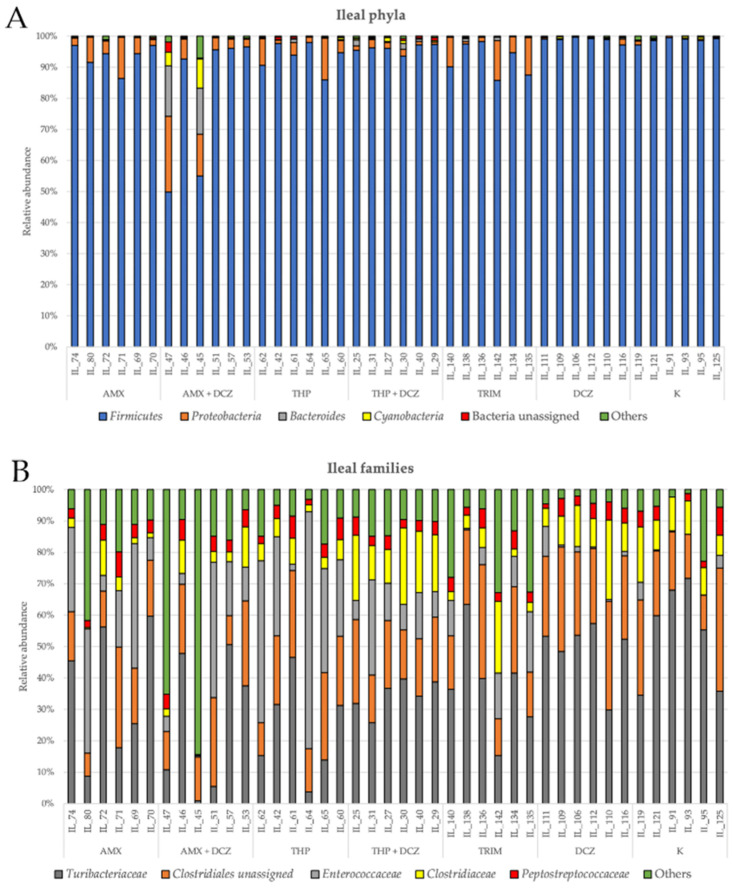
Relative abundance at the phylum (**A**) and family level (**B**) in ileum. Specific legends are reported for each bar plot.

For caecal samples, the dominant phyla were in order: *Firmicutes*, *Bacteroides*, *Proteobacteria*, an unassigned bacterial phylum, and *Cyanobacteria*. At the family level, OTUs were assigned to an unassigned family of *Clostridiales* order, *Ruminococcaceae*, *Bacteroidaceae*, *Rikinellaceae*, and *Barnesiellaceae*. Among ileal samples, the main five phyla were in order: *Firmicutes*, *Proteobacteria*, *Bacteroides*, *Cyanobacteria*, and an unassigned bacterial phylum. At the family level, the main taxa identified were, respectively: *Turibacteriaceae*, an unassigned family of *Clostridiales* order, *Enterococcaceae*, *Clostridiaceae*, and *Peptostreptococcaceae*. Relative abundances of the aforementioned taxa are shown in bar plots of Figure 3 for caecum and Figure 4 for ileum.

Two-way ANOVA was performed on the abundance of OTU values belonging to the aforementioned phyla and families. Related p values are presented in Table 1. Detailed results of the two-way ANOVA are available in Table S1.

**Figure 5:**
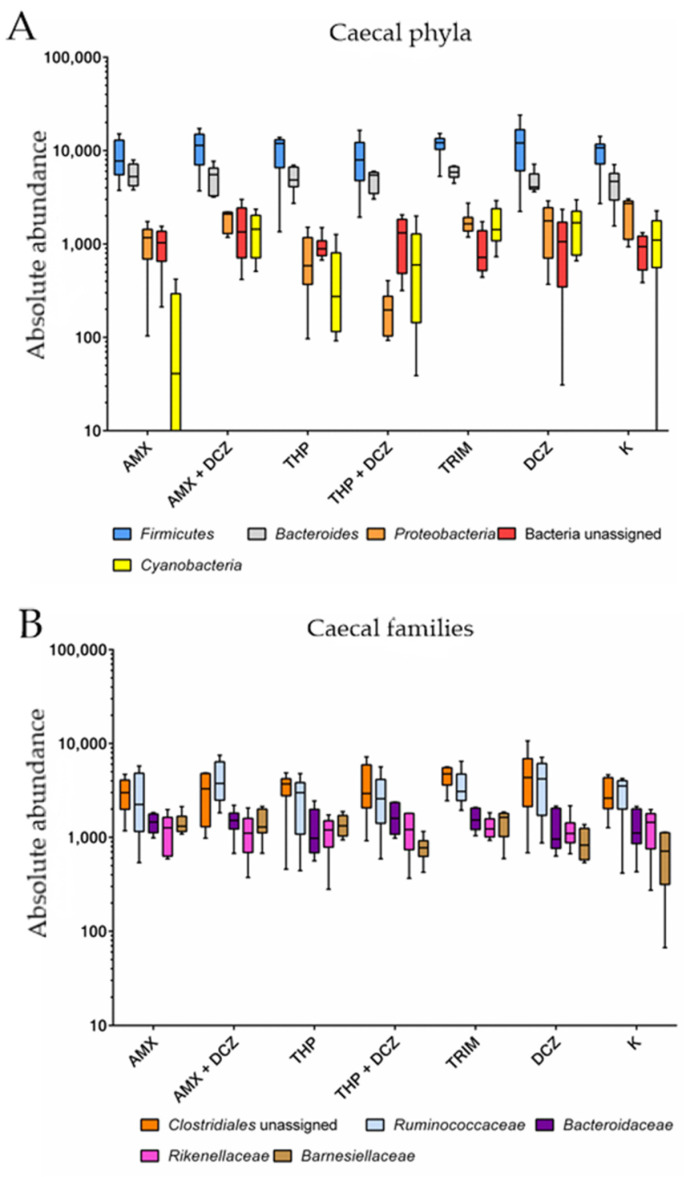
Box plots of two-way ANOVA analysis for caecal phyla (**A**) and families (**B**).

**Figure 6:**
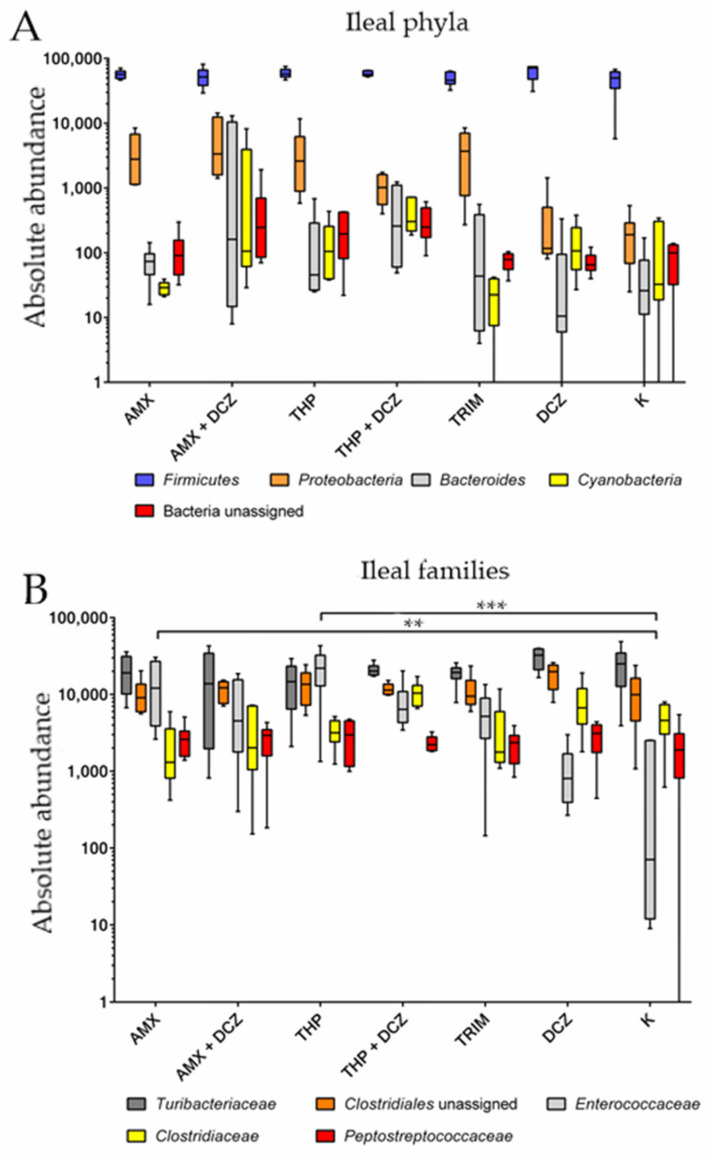
Box plots of two-way ANOVA analysis for ileal phyla (**A**) and families (**B**). (** *p*-value < 0.01; *** *p*-value < 0.001).

The results show that an effect of the studied taxa was always significant on the total variance of the samples. Box plots are shown in Figure 5 (caecum) and Figure 6 (ileum). Most intriguingly, however, in Tukey’s post-test the ileal families *Enterococcaceae* have been shown to be significantly overrepresented in our samples in amoxicillin group (AMX) (*p*-value = 0.004) and in thiamphenicol group (THP) (*p*-value < 0.0001) groups in comparison to control group K.

## 3. Discussion

In the last two decades, AM use in veterinary medicine has undergone several changes and limitations mainly considering the threat of AMR. Prophylactic and therapeutic uses of AMs are still permitted, even if their way of use has been continuously revised in compliance with national and international laws. Poultry production is particularly concerned by these changes. It is well known that broiler GM is a favorable environment for the dissemination of AMR genes between commensal and pathogenic bacteria [16]. Moreover, the selective pressure of AM treatments can enforce the selection of resistant bacteria worsening the AMR threat in poultry productions [17]. On the other hand, any modifications of GM, also known as dysbiosis, could alter broiler physiology and increase susceptibility to infectious disease, or also metabolic and inflammatory dysfunctions [2].

In our study, we aim to investigate the impact of different treatments on broiler GM in ileum and caecum. Several studies have described the microbial communities that colonized the two different intestinal tracts [7,18]. Their microbial composition reflects the different functions of these organs, and their relationship has brought mutual benefits for each other. In particular, the ileum is the main site of nutrient absorption and it is generally colonized by bacteria that influence nutrients availability and host metabolism [6,7]. For example, *Lactobacillus* spp. is one of the main represented genera and its functions have been related to the production of essential vitamins, but also the recycling of intestinal bile acids [7]. On the other side, caecum is an important site of starch fermentation, water absorption, and urea recycling and these activities are strictly dependent on the metabolic capacity of caecum microbiota [6,19]. Caecal GM has always been reported as the one with the highest richness and abundance in bacterial composition and *Bacteroides* spp., *Ruminococcus* spp., and *Faecalibacterium* spp. are the main genera that have been found [7,18,20]. Our results highlighted that different AM treatments have a diverse impact on ileal and caecal communities.

Considering alpha diversity metrics, which are indicators of the abundance of a microbial community [21], our results show significantly different Pielou’s evenness (*p* = 0.03 for ileum; *p* = 0.5 for caecum) and Faith’s phylogenetic diversity (*p* = 0.04 for ileum; *p* = 0.26 for caecum) only in ileum. These results can suggest that the withdrawal periods could be not enough for the restoral of the abundance of microbial communities in the ileum. It is well known that broiler caecum harbors the richest and most diverse microbiota in comparison with other intestinal tracts [22,23,24]. Moreover, Mohd Shaufi et colleagues [25] have shown that caecum microbiota presented a significant difference in the expression of the bacterial colonization pathway (e.g., motility proteins, two-component system, and bacterial system). All these considerations can help to explain the alpha diversity metrics not affected in caecal samples.

On the other hand, beta diversity metrics are significantly affected by AM treatment (*p* = 0.001 in the caecum and *p* = 0.002 in the ileum). Beta indexes describe compositional changes between different microbial communities [21]. As expected, the six AM protocols caused different changes in GM of ileum and caecum. These significant results can also be visualized in PCoA plots (Figure 1 and Figure 2), where caecal control samples are clearly separated from the others. Otherwise, the ileal PCoA plot shows less separation between groups probably due to a lower diversity of ileal GM than the caecal one, as mentioned above. First, we can explain this result with the different broad-spectrum action of amoxicillin, thiamphenicol, and trimethoprim–sulfadiazine. In addition, the association with diclazuril can modulate the AM activity against gut bacteria. In our case, sulfadiazine–trimethoprim is not active against bacteria belonging to *Enterococcus* and *Pseudomonas* genera [26]. On the contrary, thiamphenicol and amoxicillin have a wider antibacterial activity than sulfadiazine–trimethoprim. Amoxicillin is often associated with clavulanic acid to improve antibacterial activity against β-lactamases producers [27]. In Italy, amoxicillin is only permitted as monotherapy in poultry production, and several pieces of evidence support that broiler GM is a reservoir of different β-lactamases producing bacteria, e.g., Extended-Spectrum-Beta-Lactamases producing *Enterobacteriaceae* [28,29].

In our study, taxonomy results were analyzed by two-way ANOVA to verify if a differentially abundant taxon related to the different AM treatment groups existed. The extremely abundant phylum in ileal samples was Firmicutes, and, due to this high prevalence, the phyla significantly affected the variance in our samples both in ileum and caecum. This result is in accordance with other published works, where Firmicutes is the main phyla in ileum accounting for more than 90% of the total OTUs and also the dominant one in caecum but less frequent than other phyla [4,7]. Moreover, in ileal samples, the *Enterococcaceae* family was shown to be significantly affected by AM treatments in groups AMX and THP. The increase in bacteria belonging to this family may be due to an increased resistance against amoxicillin and thiamphenicol. *Enterococcaceae* are known to present a naturally reduced susceptibility to penicillins, due to the expression of low-affinity penicillin-binding proteins [30,31]. Resistance to phenicols, instead, has been recently described in *Enterococcus* spp. harboring multi-drug resistance plasmids isolated from both animal and human samples [30,32].

## 4. Materials and Methods

### 4.1. Animals and Samples Collection

A total of 240 male broilers (Ross 308) were reared under the same conditions in the chicken broiler farm facility of the Department of Veterinary Sciences of the University of Turin as previously reported [33]. Briefly, chicks were randomly allocated into 7 groups. During production cycles, six groups received a different antimicrobial prophylactic program: thiamphenicol (Tirsan O.S. 200 mg/g) (THP), amoxicillin (Amoxid Polv 1430 g) (AMX), sulfadiazine + trimethoprim (Trimethosulfa orale) (TRIM), thiamphenicol (Tirsan O.S. 200 mg/g) + diclazuril (Coxiril) (THP + DCZ), amoxicillin (Amoxid Polv 1430 g) + diclazuril (Coxiril) (AMX + DCZ), and diclazuril (Coxiril) (DCZ). Untreated animals were used as a control group (K). Chickens received a starter diet from 0 to 25th day and a grower diet from 26th to 58th day of rearing and AMs were administered in both these periods. Water and feed were provided ad libitum during the whole cycle. The environmental conditions (lighting programs, temperature, relative humidity, and ventilation rates) were regulated accordingly to the Ross broiler management guidelines. AMs were administered via drinking water. Detailed prophylactic protocols are available in Table 2. The withdrawal periods were respected before slaughtering. At the end of the rearing cycle (58 days), 120 broilers were regularly slaughtered, in particular, 15 animals per each treated group and 30 animals of the untreated group. Intestinal contents from ileum and caecum were aseptically collected. Samples were immediately stored at −80 °C.

### 4.2. DNA Extraction and 16S rRNA Sequencing

Genomic DNA was isolated from 6 samples, randomly selected in each group, both for ileal and caecal content. DNAzol (Thermo Fisher Scientific, Waltham, MA, USA) and DNeasy PowerClean Pro Cleanup Kit (QIAGEN, Hilden, Germany) were, respectively, used for DNA extraction and purification according to the manufacturer’s procedures. DNA was quantified using a Nanodrop spectrophotometer (Thermo Fisher Scientific, Waltham, MA, USA). Library preparation and 16S rRNA gene sequencing were performed by an external laboratory (BMR Genomics, Padova, Italy). The variable V3 and V4 regions were amplified with universal prokaryote primers Pro341F and Pro805R [34]. A Nextera XT Index kit (Illumina, San Diego, CA, USA) was used for libraries’ preparation. Finally, amplicons were sequenced with the Illumina MiSeq platform using a 2 × 300 bp paired-end protocol.

### 4.3. Bioinformatic Analyses

Raw reads were divided according to their intestinal origin (ileum and caecum) and analyzed separately with the same bioinformatic pipeline. The quality of raw reads was checked with FastQC v. 0.11.9 software. Then, raw reads were processed following Qiime2 v. 2019.10 pipeline [35]. Primers were trimmed using Cutadapt [36]. Low-quality reads with a q-score < 20 were removed. After trimming and filtering steps, clean reads were processed with DADA2 [16]. The amplicon sequence variant (ASV) table was used for generating the rooted and unrooted phylogenetic trees. Afterwards, alpha (Pielou’s evenness and Faith’s phylogenetic Diversity) and beta diversity indexes (unweighted UniFrac, weighted UniFrac, Jaccard, and Bray-Curtis distances) were determined. PCoA plots were generated using Emperor tool in Qiime2. Statistical analyses were conducted using Qiime2 plugins. A pairwise Kruskall–Wallis test and nonparametric Permutational Multivariate Analysis of Variance (PERMANOVA) tests were conducted, respectively, for alpha and beta metrics. The alpha-rarefaction curve was generated to understand if the sequencing depth was enough to describe all microbial communities. Finally, taxonomy was assigned to the obtained ASV table using the Greengenes database clustered at 99% identity. The Greengenes database was previously trained to the region targeted by sequencing primers. Box plots were generated for taxonomy results visualization.

### 4.4. Taxonomy Analyses

Data from the taxonomic assignment of OTUs were tested for differentially abundant taxa for both phyla and families. Statistical analyses were performed by two-way ANOVA using GraphPad Prism v.6 (GraphPad Software, San Diego, CA, USA). The two effects evaluated were the AM treatment group (AMX, AMX + DCZ, THP, THP + DCZ, TS, DCZ, and K) and the top five phyla and families represented in ileal and caecal samples. We tested multiple comparison corrections with Tukey’s test when two-way ANOVA showed a *p*-value < 0.05 or a significant interaction was revealed.

## 5. Conclusions

In conclusion, our study demonstrates that AM prophylactic protocols, as they are normally adopted in poultry production, are responsible for GM compositional alterations. Moreover, the respected withdrawal periods were not enough for the restoral of a GM similar to the K group’s one. This conclusion could seem to be in accordance with Elokil et al. [37], who recently published a paper where adult broilers had negative consequences on GM after enrofloxacin administration. However, this study is not comparable with ours because the age of broilers and the applied AM protocols are different. These changes to GM can lead to negative consequences for broilers, and, in particular, future studies should focus on how GM alterations affect host intestinal physiology. Moreover, further investigations focusing on the selective pressure applied by AM treatments on the expression of AMR genes in GM need to be carried out.

## Figures and Tables

**Table 1:** *p*-Values summary from two-way ANOVA analysis of the top five phyla and families in caecal and ileal samples.

	*p*-Values			
Source of Variation	Phyla		Families	
	Caecum	Ileum	Caecum	Ileum
Interaction	0.92	0.31	0.32	<0.0001
Groups	0.64	0.32	0.81	0.17
Taxa	<0.0001	<0.0001	<0.0001	<0.0001

**Table 2:** Antimicrobial (AM) prophylactic treatment protocols applied during broiler rearing. The animals were divided into seven groups and slaughtered after a withdrawal period.

Group	AMs	Dosing Protocols *per os*	Time-Lapse of Treatments (Rearing Days)	Withdrawal (Days)
AMX	Amoxicillin	30 mg·kg^−1^ b.w. twice/day	20–22/53–56	1
AMX + DCZ	Amoxicillin Diclazuril	30 mg·kg^−1^ b.w. twice/day 1 mg/kg	20–22/53–56 0–52	1 5
THP	Thiamphenicol	65 mg·kg^−1^ b.w./day	23–25/47–51	6
THP + DCZ	Thiamphenicol Diclazuril	65 mg·kg^−1^ b.w./day 1 mg/kg	23–25/47–51 0–52	6 5
TRIM	Sulfadiazine Trimethoprim	20 mg·kg^−1^ b.w./day 4 mg b.w./day	21–25/50–54	3
DCZ	Diclazuril	1 mg/kg	0–52	5
K	-	-	-	-

b.w.: body weight.

## Data Availability

The 16S rRNA sequencing raw reads presented in this study can be found online at https://www.ncbi.nlm.nih.gov/sra under the accession number PRJNA698460.

## Supplementary Materials

The following are available online at https://www.mdpi.com/2079-6382/10/2/146/s1, Table S1: two-way ANOVA results.
